# The Magnifying Effect of Marital Satisfaction on the Dyadic Effect of Disabilities on Life Satisfaction

**DOI:** 10.3390/ijerph18105352

**Published:** 2021-05-18

**Authors:** Liman-Man-Wai Li, Da Jiang

**Affiliations:** 1 Department of Psychology, The Education University of Hong Kong, Hong Kong, China; 2 Department of Special Education and Counselling, The Education University of Hong Kong, Hong Kong, China; djiang@eduhk.hk

**Keywords:** disabilities, life satisfaction, actor-partner interdependence

## Abstract

(1) Background. Extending previous work, the present study examined whether marital satisfaction would magnify the dyadic effect of disabilities on life satisfaction among older married couples. (2) Methods. With responses collected from 11,694 participants (5847 couples; *M*age = 63.36 years, median: 62 years) in a large-scale survey study in China in 2015, the actor-partner interdependence model (APIM) analyses were conducted to examine how marital satisfaction moderated the actor and partner effects of disabilities on life satisfaction. In addition, mixed linear model analyses were conducted to examine the gender effect. (3) Results. The results showed that marital satisfaction magnified the negative association between disabilities and life satisfaction with different patterns for each gender. Specifically, husbands’ disabilities significantly negatively predicted their own levels of life satisfaction among those with higher marital satisfaction but not among those with lower marital satisfaction. In contrast, for wives, spousal disabilities significantly predicted lower levels of life satisfaction among those with higher marital satisfaction but not among those with lower marital satisfaction. (4) Conclusions. The evidence for the magnifying effect of marital satisfaction obtained in the present study implicates the importance of taking dyadic dynamics in close relationships into account in health care research.

## 1. Introduction

The notion that partners in close relationships dyadically affect each other’s well-being is not new [1]. Supporting it, evidence converged to show that one’s well-being is affected by not only their own personal characteristics, such as physical and mental health conditions, but also their spousal characteristics in close relationships [2,3,4]. These findings can be more crucial for providing important insights for research on older adults, as close relationships are an important part of their social life as well as an important source of support when people get older [5]. 

Despite a surge in research on the dyadic effect of health on well-being in close relationships, further work is needed for advancing our understanding of the influence of relationship dynamics on people’s psychological well-being [2,6]. First, the role of relationship quality has been mostly neglected in the field. While many studies focused on the positive influences of marital satisfaction on one’s well-being [7], some studies suggested that having satisfying marital relationships can have its downsides when partners suffer from health-related issues [8]. However, empirical evidence for the moderating effect of marital satisfaction on the dyadic effect of health was still relatively rare. In addition, previous work was mostly restricted to a single specific health condition [6], which may fail to capture that different disabilities can share some similarities or interplay with each other in shaping people’s well-being. Therefore, examining the influence of separate types of disabilities may not lead to an accurate conclusion for older people who are likely to experience more than one disability. 

Instead of focusing on one specific health condition, the present study extended previous work by examining the association between overall disabilities across domains (e.g., physical disabilities, vision loss, and cognitive impairment) and life satisfaction. Importantly, to advance the understanding of the interdependent relationship between health condition and life satisfaction in close relationships, the present study examined the dyadic effect of overall disabilities on life satisfaction by exploring the moderating effect of marital satisfaction among older married couples, whose marital relationship assumes an important part of their social life [5]. 

### 1.1. A Dyadic Approach for Health and Well-Being in Close Relationships 

Health conditions are important for individuals’ subjective well-being. In general, poor physical and mental health conditions predict a lower level of subjective well-being [9], and this pattern can be observed across different societies, including both Western [10] and Asian societies [11]. According to the interdependence theory and actor-partner interdependence theory [12,13], in addition to one’s own health condition (i.e., the actor effect), spousal health condition can also affect one’s subjective well-being (i.e., the partner effect) because of the non-independent nature of close relationships. One reason for the dyadic effect can be due to crossover effects, in which both partners need to work together to address the challenges associated with poor health conditions of their partner [3,14]. Another reason for the dyadic effect can be due to emotional contagion [15], in which partners’ negative emotions associated with their poor health conditions may be easily transmitted to their partners through daily-life interactions. Supporting the above theorizing, previous studies found that spousal physical health conditions predicted one’s psychological well-being above and beyond their own physical health conditions [3,6]. 

Following the established theories and evidence discussed above, we expected that one’s own disabilities (i.e., the actor effect) and their spousal disabilities (the partner effect) would be negatively associated with one’s life satisfaction.

### 1.2. The Moderating Role of Marital Satisfaction

To better understand the actor and partner effects of disabilities on life satisfaction between two partners in a marriage, we explored the moderating role of one’s marital satisfaction in these examined patterns. Despite the importance of marital satisfaction on mental and physical health, little is known about its moderating role [8]. 

Different from its generally positive direct effect on well-being [7], some studies considering the moderating role of marital satisfaction revealed that it can backfire in some health situations [16,17,18]. For instance, Santos et al. found a negative association of symptoms of anxiety and quality of life only among patients with multiple sclerosis reporting higher marital satisfaction [19]. However, to our knowledge, there were no studies examining the moderating role of marital satisfaction on both actor and partner effects of disabilities simultaneously on life satisfaction. Following earlier findings, the magnifying effect of marital satisfaction on the negative association between disabilities and life satisfaction is possibly observed for both actor and partner effects. On the one hand, the spouse is likely to be the primary caregiver for people with disabilities, especially among older adults [20]. On the other hand, it is common that caregivers perceive a great burden when providing support to patients with disabilities [21]. Therefore, not only the person with disabilities but also their family caregivers experience a tremendous amount of stress associated with the disability conditions. Monin et al. also found that people experienced distress when seeing other people suffering from pain, and this pattern was more notable when seeing their partners suffering [18]. Therefore, it may be likely that one’s life satisfaction can be significantly dampened when they witness their partners suffering from taking care of them (i.e., the actor effect) or when they witness their partners suffering from disabilities (i.e., the partner effect). These patterns could be more notable when people are in a satisfying marriage. 

Taken together, these considerations supported our expectation of a magnified moderating effect from marital satisfaction for the actor and partner effects of disabilities on one’s life satisfaction. Specifically, we expected that a stronger negative association between one’s disabilities and one’s life satisfaction (i.e., the actor effect) and a stronger negative association between spousal disabilities and one’s life satisfaction (i.e., the partner effect) would be found among people with higher levels of marital satisfaction than those with lower levels of marital satisfaction. 

### 1.3. The Moderating Role of Gender 

One frequently examined influence on the actor-partner interdependent effect in close relationships is the gender effect. Theories of traditional gender roles proposed that the partner effect would be stronger for women in the health context. Compared with men, women are likely to take the role of caregivers and provide more support in the family context [22], which can cause significant stress for women [21]. In addition, women are found to be more sensitive to the needs of their partners because of their stronger relational orientation [23]. 

However, mixed findings regarding the gender effect have been observed. Ayotee et al. found that wives’ depressive symptoms were more likely to be affected by husbands’ health conditions, while there was no reliable evidence showing that husbands’ depressive symptoms were affected by their wives [2]. Their findings were consistent with the gender norm perspectives. In contrast, Ruthig et al. found that husbands’ well-being was significantly predicted by their wives’ health conditions, while wives’ well-being was not significantly predicted by their husbands’ health conditions [6]. The authors argued that while older women can get support and companionship from their larger, well-established social networks, older men are highly dependent on their wives, which can possibly lead to a more notable influence of wives’ health conditions on husbands’ well-being. Bourassa et al. did not find gender differences in the strength of the association between spousal health and one’s own quality of life [3]. They speculated that gender differences in the partner effect may be stronger when caregiving is strongly needed for more severe clinical illnesses. Because of the inconsistent (and limited) findings, we did not form specific hypotheses while exploring the effect of gender in the analyses. 

Based on the above speculations, we tested the following hypotheses in the present study:

**Hypothesis** **1** **(H1).** *One’s own disabilities (i.e., the actor effect) and their spousal disabilities (the partner effect) are negatively associated with one’s life satisfaction.*

**Hypothesis** **2** **(H2).** *A stronger negative association between one’s disabilities and one’s life satisfaction (i.e., the actor effect) and a stronger negative association between spousal disabilities and one’s life satisfaction (i.e., the partner effect) are found among people with higher marital satisfaction than those with lower marital satisfaction.*

We also explored the moderating role of gender. Given the inconsistent findings, we did not form a specific hypothesis for the gender effect. 

## 2. Materials and Methods

### 2.1. Participants

To examine the role of marital satisfaction in moderating the dyadic effect of overall disabilities on life satisfaction among older married couples, we used the data from China Health and Retirement Longitudinal Study (CHARLS). The data were collected via interviews with a nationally representative sample of adults and their spouses in China using multistage cluster sampling [24]. CHARLS collected data in 2011, 2013, 2015, and 2018. The data collected in 2015 (CHARLS 2015) were used in the present study. This dataset was the latest available dataset with relevant variables when we started this research project. 

The sampling selection procedure was listed as follows. A total of 21,095 participants were found in the dataset of CHARLS 2015. We adopted the following screening criteria to select the married older couples in the final analyses: 1) both partners completed the study (*n* = 17,670 participants); 2) both partners reported their gender (*n* = 17,538 participants); 3) participants reported that they were married and living together with their partner (*n* = 16,128 participants); and 4) the age of both partners was 50 years or above. There were 11,694 participants (i.e., 5847 couples) in the final dataset. The age of these participants ranged from 50 to 99 years, *M*age = 63.36 years, *SD* = 8.14 years, and the majority of participants lived in rural areas (rural areas: 71%; urban areas: 14%; suburban areas: 15%). All were heterosexual marriages. 

### 2.2. Measures

Disabilities. Participants indicated whether they had the following disabilities (1 = yes, 0 = no): (1) physical disabilities (*n* = 348, 3% of participants reported that they had this disability), (2) brain damage/cognitive impairment (*n* = 317, 3%), (3) vision problems (*n* = 540; 5%); (4) hearing problems (*n* = 756; 7%), and 5) speech impediments (*n* = 72; 1%). The sum score was used as an indicator for overall disabilities (*M* = 0.17, *SD* = 0.46, range: 0–5).

Life satisfaction. Participants were asked to indicate how satisfied they were with life, their life-as-a-whole, on a 5-point scale ranging from 1 (completely satisfied) to 5 (not at all satisfied). The score was reversed in the final analysis, with a greater number indicating a higher level of life satisfaction (*M* = 3.42, *SD* = 0.76, range: 1–5).

Marital satisfaction. Participants indicated their marital satisfaction on a 5-point scale ranging from 1 (completely satisfied) to 5 (not at all satisfied). The score was reversed in the final analysis, with a greater number indicating a higher level of marital satisfaction (*M* = 3.47, *SD* = 0.80, range: 1–5).

Control variables. The following variables were treated as control variables in the final analyses: participants’ living regions (1 = rural areas; 0 = non-rural areas), age, retirement status (1 = yes, 0 = no), and monetary resources (i.e., their savings).

### 2.3. Analytic Plan

To capture the non-independent nature of the responses of married couples, actor-partner interdependence model (APIM) analyses were conducted by following the recommendation of Kenny and Ledermann [25]. The responses of husbands and wives were analyzed in 1 model with 2 separate intercepts. In other words, the actor effect, i.e., the effect of one’s overall disabilities on their own life satisfaction, and the partner effect, i.e., the effect of their spouse’s overall disabilities on their own life satisfaction, were tested simultaneously in 1 single APIM analysis. Responses with missing data were excluded from the analyses.

We first tested the actor and partner effects of overall disabilities on one’s life satisfaction among older married couples. Next, we tested the moderating effect of one’s marital satisfaction on the above-mentioned dyadic effects of disabilities on one’s life satisfaction.

To test whether gender has a significant effect on the actor and partner effects observed in the APIM analyses, we conducted mixed linear model analyses with random intercepts and random slopes specified.

All analyses were conducted with controls for the effect of living regions, age, retirement status, and savings. All continuous independent variables (i.e., disabilities, marital satisfaction, age, and savings) were mean-centered. The APIM and mixed linear model analyses were conducted using R packages, including [nlme], [tidyr], and [dplyr]. The raw dataset can be requested online directly from CHARLS (http://charls.pku.edu.cn/index/zh-cn.html (accessed on 15 June 2020)) and the R scripts will be provided by the authors upon request.

## 3. Results

### 3.1. The Dyadic Effects of Overall Disabilities on Life Satisfaction

Table 1 presents the APIM results of the dyadic relationship between disabilities and life satisfaction.

**Figure 1:**
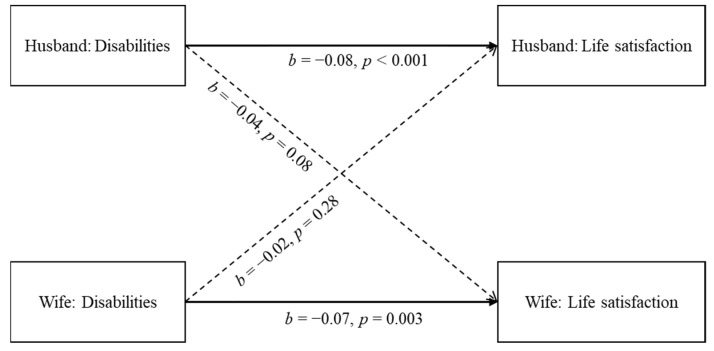
The results of APIM for the dyadic effect of overall disabilities on life satisfaction.

The results showed that the actor effects for husbands and wives were both significant, with higher overall disability scores significantly predicting lower levels of life satisfaction (husbands: b = −0.08, *p* < 0.001, 95% CI = (−0.12, −0.03); wives: b = −0.07, *p* = 0.003, 95% CI = (−0.12, −0.03)). In contrast, the partner effects were non-significant (husbands: b = −0.02, *p* = 0.28, 95% CI = (−0.07, 0.02); wives: b = −0.04, *p* = 0.08, 95% CI = (−0.09, 0.01)) (see Figure 1). Therefore, H1 was only partially supported.

For the gender effect, the mixed linear model analysis indicated that the actor effect (i.e., the association between one’s disabilities and life satisfaction), b = −0.001, *p* = 0.97, 95% CI = (−0.03, 0.03), and the partner effect (i.e., the association between spousal disabilities and one’s life satisfaction), b = 0.01, *p* = 0.59, 95% CI = (−0.02, 0.04), were not significantly moderated by gender.

### 3.2. The Moderating Effect of Marital Satisfaction on the Dyadic Effects of Overall Disabilities on Life Satisfaction

Table 2 presents the results of the APIM analyses for the moderating role of marital satisfaction on the dyadic pattern between overall disabilities and life satisfaction.

Only the moderating effect is discussed in the main text. Refer to Table 2 for the effect of other variables in the analysis. For husbands, the association between their own disabilities and life satisfaction (i.e., the actor effect) was significantly moderated by their own marital satisfaction, b = −0.09, *p* < 0.001, 95% CI = (−0.14, −0.04), while the association between their spousal disabilities and their own life satisfaction (i.e., the partner effect) was not significantly moderated by their marital satisfaction, b = 0.02, *p* = 0.36, 95% CI = (−0.03, 0.07).

The follow-up analyses showed that husbands’ disabilities were significantly negatively associated with their own life satisfaction among people with higher marital satisfaction (1SD above the mean), b = −0.12, *p* < 0.001, 95% CI = (−0.18, −0.07), but this pattern was not significant among those with lower marital satisfaction (1SD below the mean), b = 0.02, *p* = 0.49, 95% CI = (−0.04, 0.08).

For wives, the association between their own disabilities and life satisfaction (i.e., the actor effect) was not significantly moderated by their marital satisfaction, b = 0.01, *p* = 0.62, 95% CI = (−0.04, 0.06), while the association between their spousal disabilities and their own life satisfaction (i.e., the partner effect) was significantly moderated by their marital satisfaction, b = −0.06, *p* = 0.007, 95% CI = (−0.11, −0.02).

The follow-up analyses showed that husbands’ disabilities were significantly negatively associated with wives’ life satisfaction among wives with higher marital satisfaction, b = −0.08, *p* = 0.007, 95% CI = (−0.15, −0.02), but this pattern was not significant among those with lower marital satisfaction, b = 0.02, *p* = 0.551, 95% CI = (−0.03, 0.07). Thus, only the patterns obtained from husbands provided support for H2.

Regarding the gender effect, consistent with the APIM results, the mixed linear model analysis supported the significant moderating effect of gender. The results showed that the three-way interaction of gender, actors’ disabilities, and actors’ marital satisfaction was significant, b = −0.05, *p* = 0.004, 95% CI = (−0.09, −0.02), and the three-way interaction of gender, partners’ disabilities, and actors’ marital satisfaction was also significant, b = 0.04, *p* = 0.01, 95% CI = (0.01, 0.08).

## 4. Discussion

The results showed that, in general, one’s disabilities but not their spousal disabilities significantly predicted their own life satisfaction. Importantly, when we considered the moderating role of one’s marital satisfaction, the results were more complex—husbands’ disabilities were negatively associated with their own life satisfaction only when they had a higher level of marital satisfaction, and husbands’ disabilities were negatively associated with their wives’ life satisfaction only when the wives had a higher level of marital satisfaction. In short, marital satisfaction magnified the negative influence of disabilities on life satisfaction for both actor effect (for husbands only) and partner effect (for wives only).

These findings can bring important implications for advancing the understanding of the dyadic effects of physical and mental health on people’s life satisfaction in close relationships. Previous work reported some inconsistent findings regarding the actor and partner effects of health in close relationships [2,3,6]. One possible reason for the inconsistency in the literature could be because of a neglect of marital relationship quality in previous work. Although the interdependence theory and actor-partner interdependence theory [12,13] posit that it is important to consider the influence of shared social contexts between couples, many studies did not carefully consider the quality of shared social relationships in affecting the dyadic effects [8]. Despite the overall positive influence of marital satisfaction in diverse domains [7], the present study demonstrated that, instead of buffering the negative influence, greater marital satisfaction magnified the negative dyadic influence of health on life satisfaction in older married couples, which was consistent with some previous work [4,19]. For instance, Polenick et al. found that relationship closeness magnified the negative association between patients’ illness severity and spouses’ well-being [4]. Consistent with other types of social relationships such as friendships [26], the quality of a given relationship exerts a significant moderating effect in shaping people’s psychological functioning and behaviors in their social life. These findings suggest that we should consider not only the shared nature of a relationship but also the quality of the given relationship (e.g., satisfaction and closeness) for a comprehensive understanding of the role of relationship dynamics in health-related scenarios, such as health care settings.

The present study also discovered some interesting findings regarding the gender effect when we considered the moderating role of marital satisfaction. The results showed that greater marital satisfaction magnified the negative association between their own disabilities and life satisfaction for husbands only and magnified the negative association between their spousal disabilities and their own life satisfaction for wives only.

The asymmetric patterns for men and women observed in the present study might be explained by the expectations of gender roles in the family setting. Given that wives would be more likely to take the caregiving role [20], they are expected to provide assistance and support for their husbands, especially when their spouses are with disabilities. In contrast, adult children or relatives instead of their husbands would be more likely to take the caregiving role when wives are with disabilities [27]. Because of the differences in the expected family roles, marital satisfaction may magnify the negative effect of their own disabilities on their life satisfaction for husbands, as the husbands may feel remorse for causing a heavy burden to their wives, and this feeling can be stronger for those satisfied with their marital relationships. In contrast, husbands who are dissatisfied with their marital relationships may not have high expectations from their wives. Yet, wives may be still likely to provide good care and support to their husbands regardless of their marital relationship quality because of the strong gender role expectation for women [20,27]. The unexpected care provided by their wives might weaken, or even eliminate, the effect of their disabilities on life satisfaction among husbands. The gender role norms cause different experiences among women. Marital satisfaction may magnify the negative effect of husbands’ disabilities on wives’ life satisfaction, as the wives are the primary caregivers that are likely to be under a great amount of stress [20,27]. In addition, wives’ stronger relational interdependence [23] may cause them more pain when they witness their partners suffering from disabilities [18] in a satisfying marital relationship. As discussed in the introduction, the moderating effect of marital satisfaction has not been fully examined in the existing literature [8]. Further work is needed for advancing our understanding of the moderating effect of marital satisfaction between men and women.

Some practical implications can be made based on the present findings. Consistent with previous work [3,28], the present study provides further evidence to support the view that treatment should not only focus on patients but also extend to provide support to their partners in close relationships. That means dyadic interventions should be used [29]. Although it was not the focus in the present study, we found that lower levels of marital satisfaction predicted lower levels of life satisfaction among both men and women, which was consistent with previous studies [7]. Dyadic interventions may help promote and maintain high-quality relationships, resulting in greater mutual support between the two partners [5] and thus promoting better well-being. For instance, in addition to providing appropriate medical treatment to patients, we could have spouse-assisted training to equip partners with skills and knowledge for caregiving, which may help reduce patients’ psychological distress [30]. Additionally, some studies found that personal and spousal resilience both contributed to better well-being among older married couples in China [31], which suggests that we could provide training to foster resilience of both partners using positive psychology interventions. Furthermore, some studies revealed that personal and community-related factors jointly affect the level of well-being among Chinese older adults [32], suggesting the importance of community-based services for older adults with disabilities. As suggested in previous studies, social services can be provided to spousal caregivers [33], and community-based centers can be established to provide a wide range of services, including both medical care and emotional support from the community, to older married couples with disabilities [34].

Importantly, the present study further suggested that we should not ignore the needs of people who have highly satisfying marital relationships. The present findings showed magnifying effects of marital satisfaction on both actor and partner effects of disabilities on life satisfaction. These results suggested that dyadic interventions and community-based services may be particularly crucial for people in a highly satisfying marital relationship, as they are more likely to experience pain while seeing their partners suffering from disabilities or diseases [18]. Given that family income was found to be positively associated with marital satisfaction in China [35], the level of marital satisfaction may have been further improved from 2015 to recent years due to an increase in family income associated with economic growth. The findings of the present study might urge a stronger call to examine the potential negative effects of marital satisfaction on older married couples with health-related challenges in more recent years.

We acknowledge some limitations in the present study. First, the present correlational data could not determine the causal relationship between disabilities and life satisfaction, though some longitudinal evidence suggested that life satisfaction was the consequence of poor physical health, rather than vice versa [9]. Experiments are needed to test the causal relationships among examined variables. Second, single items were used for marital satisfaction and life satisfaction, which did not allow us to test the reliability of the adopted measures. However, this concern can be minimal as some studies provided evidence for the high validity of using single items [36]. Regardless, future studies need to adopt full-item scales for marital satisfaction and life satisfaction to ensure validity and reliability of the adopted measurements. Using the full-item scales may provide opportunities to explore which domains of marital quality (e.g., communication or conflicts) may exert stronger moderating effects on the dyadic effects of disabilities on life satisfaction among older married couples. Third, the present study examined the patterns among older participants. Compared with other age groups, close relationships could matter more to older participants, as they have more interactions with their partners than people in other types of social relationships [5]. Future studies are needed to explore whether the patterns would be generalizable to different age groups that may prioritize close relationships differently.

## 5. Conclusions

While the positive role of marital satisfaction is commonly accepted, the present study revealed that marital satisfaction can backfire, that is, higher levels of marital satisfaction can strengthen the negative association between one’s disabilities and their own life satisfaction among men as well as the negative association between spousal disabilities and their own life satisfaction among women. These findings highlight the importance of taking the dyadic dynamics, such as relationship quality, in close relationships into account for a better understanding of the influence of health on people’s well-being.

## Figures and Tables

**Table 1:** The results of APIM for the dyadic effect of overall disabilities on life satisfaction.

APIM Parameters	Role	Estimate	SE	95% CI
	Husband			
Intercept		3.46 ***	0.24	3.41, 3.51
Region (rural = 1; non-rural = 0)		−0.03	0.03	−0.08, 0.02
Age		0.003 *	0.001	0.0004, 0.01
Savings		0.000001 **	0.0000002	2.75^−7^, 1.09^−6^
Retirement (yes = 1; no = 0)		0.04	0.03	−0.11, 0.02
Disability (actor effect)		−0.08 ***	0.02	−0.12, −0.03
Wife’s disabilities (partner effect)		−0.02	0.02	−0.07, 0.02
	Wife			
Intercept		3.39 ***	0.027	3.34, 3.45
Region (rural = 1; non-rural = 0)		−0.01	0.03	−0.07, 0.05
Age		0.003	0.001	−0.0002, 0.01
Savings		0.000001 **	0.0000003	3.16^−7^, 1.42^−6^
Retirement (yes = 1; no = 0)		−0.002	0.04	−0.08, 0.08
Disability (actor effect)		−0.07 **	0.03	−0.12, −0.03
Husband’s disabilities (partner effect)		−0.04	0.02	−0.09, 0.01

Note. * *p* < 0.05; ** *p* < 0.01; *** *p* < 0.001.

**Table 2:** The results of APIM for the moderating role of marital satisfaction on the dyadic effect of overall disabilities on life satisfaction.

APIM Parameters	Role	Estimate	SE	95% CI
	Husband			
Intercept		3.39 ***	0.02	3.35, 3.43
Region (rural = 1; non-rural = 0)		0.01	0.02	−0.05, 0.004
Age		0.004 ***	0.001	0.002, 0.01
Savings		0.000001 ***	0.0000002	2.56^−7^, 9.93^−7^
Retirement (yes = 1; no = 0)		−0.02	0.03	−0.08, 0.04
Marital satisfaction		0.43 ***	0.01	0.40, 0.45
Disabilities (actor effect)		−0.05 *	0.02	−0.09, −0.01
Wife’s disabilities (partner effect)		−0.002	0.02	−0.04, 0.04
Marital satisfaction * Disabilities (actor effect)		−0.09 ***	0.03	−0.14, −0.04
Marital satisfaction * Wife’s disabilities (partner effect)		0.02	0.03	−0.03, 0.07
	Wife			
Intercept		3.44 ***	0.02	3.40, 3.49
Region (rural = 1; non-rural = 0)		0.01	0.03	−0.04, 0.06
Age		0.004 **	0.001	0.001, 0.01
Savings		0.000001 **	0.0000003	1.80^−7^, 1.17^−7^
Retirement (yes = 1; no = 0)		−0.01	0.04	−0.08, 0.01
Marital satisfaction		0.42 ***	0.01	0.40, 0.43
Disabilities (actor effect)		−0.05 *	0.02	−0.09, −0.002
Husband’s disabilities (partner effect)		−0.03	0.02	−0.08, 0.01
Marital satisfaction * Disabilities (actor effect)		0.01	0.02	−0.04, 0.06
Marital satisfaction * Husband’s disabilities (partner effect)		−0.06 **	0.02	−0.11, −0.02

Note. * *p* < 0.05; ** *p* < 0.01; *** *p* < 0.001.

## Data Availability

The raw dataset can be requested online directly from CHARLS (http://charls.pku.edu.cn/index/zh-cn.html (accessed on 15 June 2020)). The present study only covered some of the measures from the 2015 wave. The research questions tested in the present study have not been investigated in previous work using this dataset.
